# Supplementary figures and images for: How urban environment shapes EV charging experience in Travis County, Texas
Source: PLoS One. 2026 Jun 2;21(6):e0349619. doi: 10.1371/journal.pone.0349619 (PMC13229328; doi:10.1371/journal.pone.0349619)

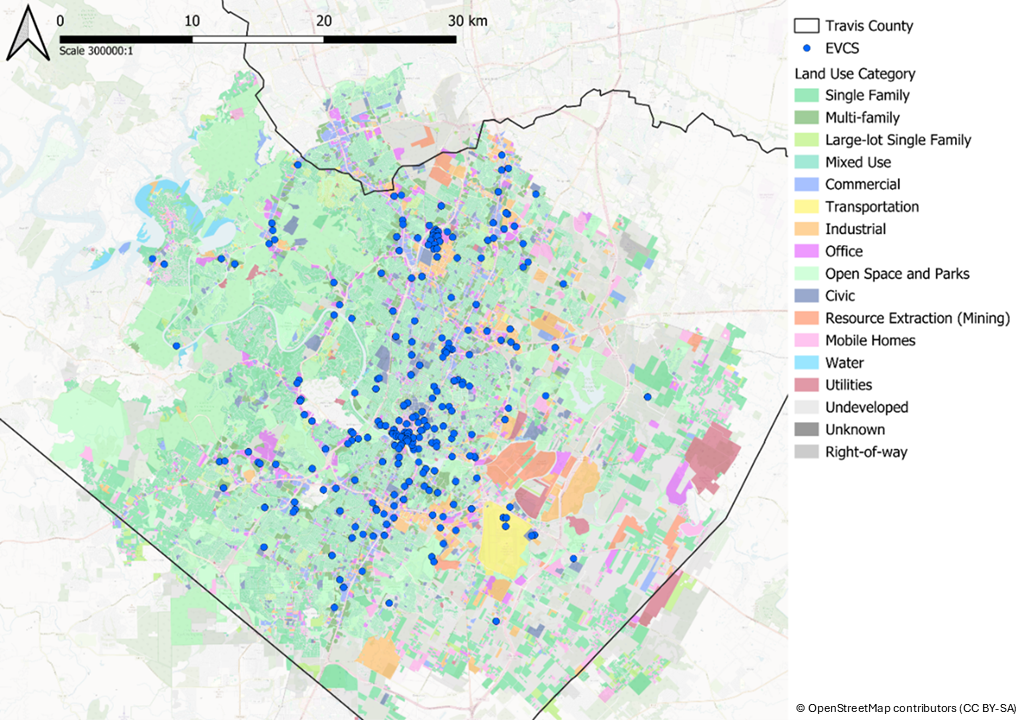

Supplement: S1 Fig — ). (PNG) [file pone.0349619.s001.PNG]

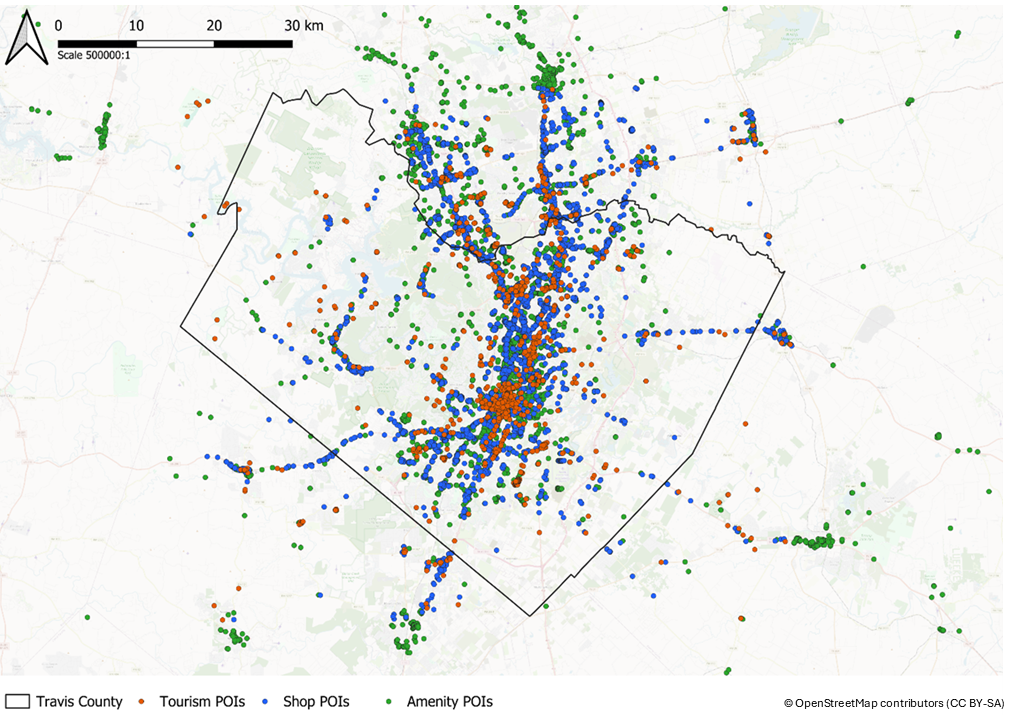

Supplement: S2 Fig — ). (PNG) [file pone.0349619.s002.PNG]

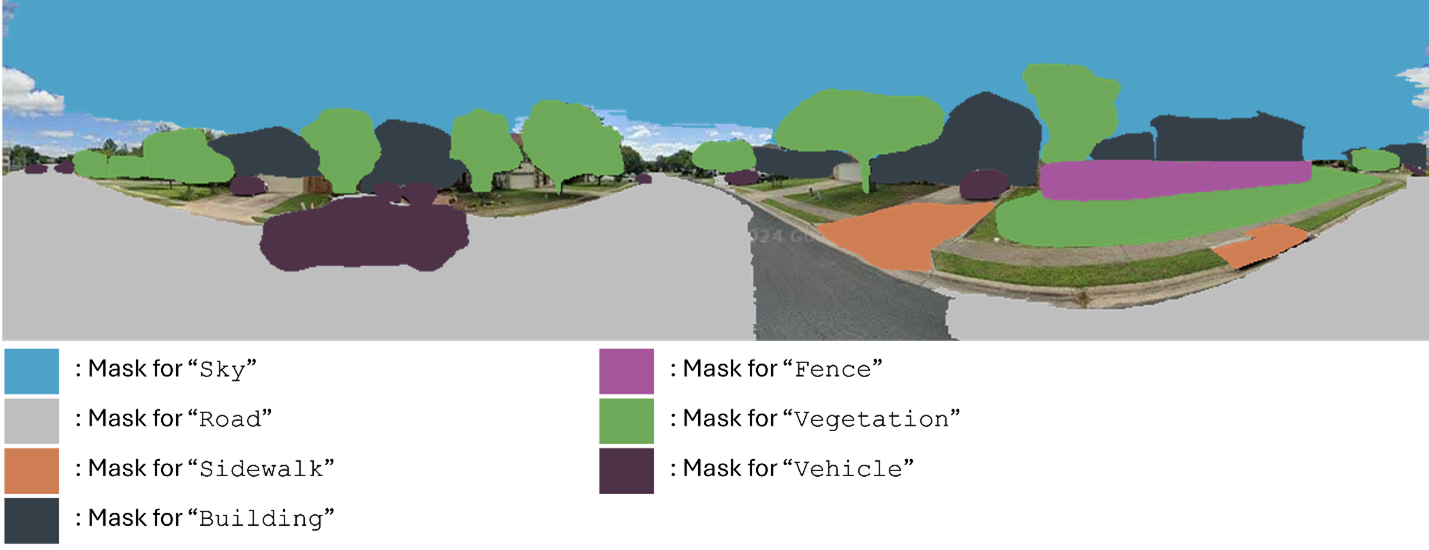

Supplement: S3 Fig — (PNG) [file pone.0349619.s003.png]

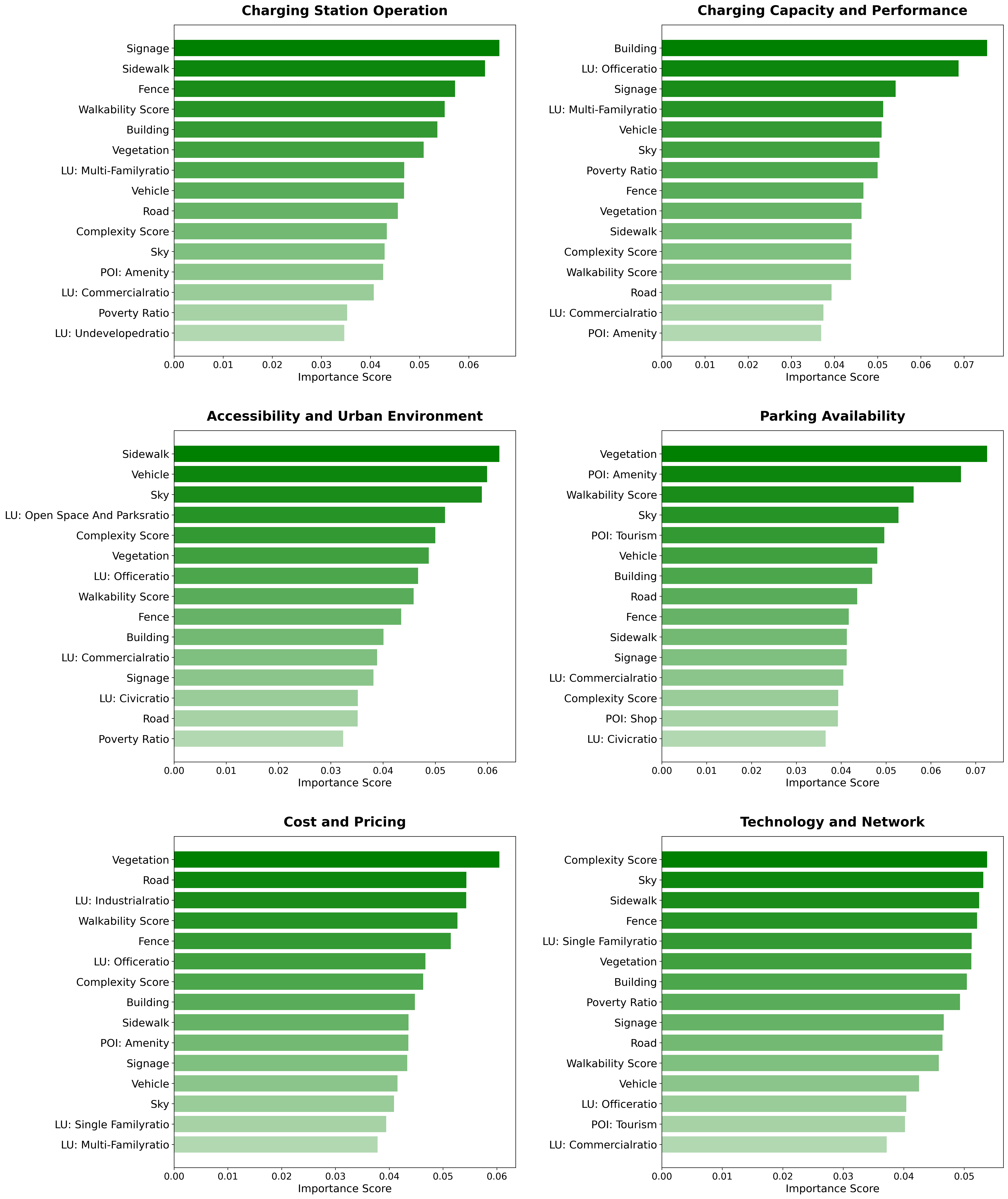

Supplement: S4 Fig — (PNG) [file pone.0349619.s004.png]
