# Supplementary material for: How urban environment shapes EV charging experience in Travis County, Texas
Source: PLoS One. 2026 Jun 2;21(6):e0349619. doi: 10.1371/journal.pone.0349619 (PMC13229328; doi:10.1371/journal.pone.0349619)
Supplement: S1 Table — (DOCX) [file pone.0349619.s005.docx]

**S1 Table** Prompt Design for Sentiment Analysis

| Analysis Task | Prompt Structure / Criteria |
| --- | --- |
| Sentiment Analysis | Prompt: "Analyze the following EV charging station review and determine its sentiment: Review: '{review}'  - If the sentiment is positive, return '1'  - If the sentiment is negative, return '0'  - If the sentiment is neutral/mixed/unclear, return '9'  Respond with ONLY the number ('1', '0', or '9') and no extra text." |
| Category Classification (Base Prompt) | Prompt: "Review: '{review}'  {category_prompt}  - If YES, return '1'  - If NO, return '0'  Respond with ONLY the number ('1' or '0') and no extra text." |
| - Charging Station Operation | {category_prompt}: "Does this review mention station availability, reliability, or functionality?" |
| - Charging Capacity and Performance | {category_prompt}: "Does this review discuss charging speed, power, or performance?" |
| - Accessibility and Urban Environment | {category_prompt}: "Does this review mention ease of access, signage, or urban design?" |
| - Parking Availability | {category_prompt}: "Does this review mention parking issues, availability, or ease of use?" |
| - Cost and Pricing | {category_prompt}: "Does this review discuss pricing, fees, or value for money?" |
| - Technology and Network | {category_prompt}: "Does this review mention network reliability, mobile apps, or RFID systems?" |
| - Others | {category_prompt}: "If this review does not fit into any of the above categories, classify it here." |
