# Supplementary material for: How urban environment shapes EV charging experience in Travis County, Texas
Source: PLoS One. 2026 Jun 2;21(6):e0349619. doi: 10.1371/journal.pone.0349619 (PMC13229328; doi:10.1371/journal.pone.0349619)
Supplement: S2 Table — (DOCX) [file pone.0349619.s006.docx]

**S2 Table** Parcel-level Land Use Categories

| **Land Use** | **Description** |
| --- | --- |
| Single Family | One dwelling or duplex structures in detached buildings, usually on one parcel. Includes manufactured, and non-mobile homes. |
| Large-lot Single Family | One dwelling in one building on a parcel ten acres or greater, usually used as a farm or ranch. In some cases these are redeveloped, and therefore grouped with undeveloped. |
| Mobile Homes | One or many dwellings in single buildings, designed to be mobile. |
| Multi-family | Tri and four-plexes, units with 5 or more dwellings, group housing, or retirement housing. |
| Commercial | Wholesale and retail trade and services. Includes trade of most durable and non-durable goods, building, hardware, garden, general retail merchandise, lumber, grocery, food sales, auto vehicle and gasoline sales, apparel and accessory stores, home furniture and equipment, eating and drinking, commercial art and craft studios, lodging hotels and motels, personal services, mini-warehousing and personal storage, automotive repair, automotive services, entertainment and recreation services, business services, commercial sports recreation and exercise, and amusement services. |
| Mixed Use | One building containing both commercial and residential uses. |
| Office | Includes accounting, architectural services, design services, engineering, insurance, law offices, organization/association's office, personnel, property management, real estate, secretarial services, telephone answering services, television/film/sound recording studios, travel agency, financial services, banks, savings and loans, credit unions, blood banks, treatment, and guidance centers, doctor, dental, psychological, and other medical offices, electronic, pharmaceutical, chemical, and other research and development services. |
| Industrial | Basic, light, and custom industry and manufacturing, industrial arts and crafts, general, limited and commercial warehousing and distribution, not including mini-warehousing or personal storage, heavy equipment sales and services, including automobiles and recreation vehicles, pool services, cans, paper, plastic, auto and junk recycling facilities, stables, kennels, pet services, and slaughterhouses. Quarries and oil and gas drilling facilities. Processing and storage of garbage and other wastes. |
| Civic | Half-way houses, housing for mentally and psychologically handicapped; Police stations, fire houses, post offices, jails, prisons, military installations; Day care, primary and secondary education, colleges, universities, business trade schools. Club or lodge halls, religious assembly, convention centers, museums, and libraries. |
| Open Space and Parks | Open spaces and parks set aside for environmental protection, recreation or drainage. Private and public golf courses and driving ranges. Private and public camp grounds for temporary use. Does not include permanent and semi-permanent RV parks. Areas set aside for common use, typically privately owned areas that serve as drainage but are not registered as such by public agencies. Open spaces set aside for preservation or protection. |
| Transportation | Railroad stations and right-of-way. Bus stations and other transportation facilities not used for aviation, railroads, or marinas. Airports and aviation facilities. Commercial and private marinas. Surface parking for a variety of establishments/parcels, including actual parking garage facilities or pay-for-parking parcels. Parcels on separate parcels that serve only one establishment are coded with the use of that establishment. |
| Right-of-way | Public and private areas mainly containing roadways, railways, and miscellaneous infrastructure. |
| Resource Extraction | Quarries and oil and gas drilling facilities |
| Utilities | Electric, water, and wastewater utilities |
| Undeveloped | Parcels without structures or improvements. May be developed depending on future conditions. Parcels that are predominantly used for either crops, livestock, animal husbandry, or other farmland. Also may be developed depending on conditions. |
| Water | Areas permanently submerged in water |
| Unknown | Parcels where the land use can not be determined from available sources |
