# Supplementary material for: How urban environment shapes EV charging experience in Travis County, Texas
Source: PLoS One. 2026 Jun 2;21(6):e0349619. doi: 10.1371/journal.pone.0349619 (PMC13229328; doi:10.1371/journal.pone.0349619)
Supplement: S3 Table — (DOCX) [file pone.0349619.s007.docx]

**S3 Table** Summary of Input Data

| **Feature** | **Statistics** | | | | | **Description** | **Unit** |
| --- | --- | --- | --- | --- | --- | --- | --- |
|  | **Min** | **Mean** | **Median** | **Max** | **Std. Dev.** |  |  |
| POI Count Amenity | - | 13.85 | 6.00 | 124.00 | 22.39 | Count of Amenity POIs on OSM | Count |
| POI Count Shop | - | 8.64 | 3.00 | 80.00 | 14.33 | Count of Shop POIs on OSM | Count |
| POI Count Tourism | - | 2.65 | 1.00 | 35.00 | 5.30 | Count of Tourism POIs on OSM | Count |
| Mean VI Vegetation | 84,268.40 | 346,151.91 | 296,752.67 | 1,557,553.75 | 184,817.17 | Mean Area of Semantic Segmentation Mask for Vegetation | Pixels |
| Mean VI sky | 78,133.25 | 320,453.97 | 252,379.50 | 1,235,961.00 | 217,550.68 | Mean Area of Semantic Segmentation Mask for Sky | Pixels |
| Mean VI Road | 92,719.00 | 378,111.25 | 292,455.00 | 1,504,054.67 | 226,241.30 | Mean Area of Semantic Segmentation Mask for Road | Pixels |
| Mean VI Road | - | 166,780.66 | 107,513.33 | 799,563.33 | 154,283.90 | Mean Area of Semantic Segmentation Mask for Buildings | Pixels |
| Mean VI Signage | - | 3,575.40 | 2,692.67 | 22,059.33 | 3,418.42 | Mean Area of Semantic Segmentation Mask for Signage | Pixels |
| Mean VI Person | - | 251.52 | - | 4,216.50 | 530.80 | Mean Area of Semantic Segmentation Mask for People | Pixels |
| Mean VI Vehicle | - | 40,748.71 | 27,552.50 | 271,477.00 | 42,642.49 | Mean Area of Semantic Segmentation Mask for Vehicles | Pixels |
| Mean VI Sidewalk | - | 70,935.63 | 54,523.00 | 382,251.67 | 59,253.05 | Mean Area of Semantic Segmentation Mask for Sidewalk | Pixels |
| Mean VI Fence | - | 11,196.56 | 5,708.50 | 137,690.33 | 18,271.74 | Mean Area of Semantic Segmentation Mask for Fence | Pixels |
| Mean VI Bench | - | 133.92 | - | 6,796.00 | 734.05 | Mean Area of Semantic Segmentation Mask for Benches | Pixels |
| Mean Complexity | 0.24 | 1.05 | 0.96 | 4.59 | 0.55 | Mean Complexity Score (Ma et al., 2025) | - |
| Mean Walkability | 0.00 | 0.46 | 0.24 | 5.01 | 0.80 | Mean Walkability Score (Ma et al., 2025) | - |
| Ratio Under 2x Poverty | - | 0.21 | 0.20 | 1.00 | 0.14 | The ratio of residents who live below 200% of poverty line | - |
| LU Ratio Civic | - | 0.05 | 0.00 | 0.60 | 0.09 | Aggregated surface area of civic land use | - |
| LU Ratio Commercial | - | 0.13 | 0.10 | 0.71 | 0.15 | Aggregated surface area of commercial land use | - |
| LU Ratio Industrial | - | 0.03 | - | 0.55 | 0.07 | Aggregated surface area of industrial land use | - |
| LU Ratio Large-lot Single Family | - | 0.00 | - | 0.30 | 0.01 | Aggregated surface area of large-lot single family land use | - |
| LU Ratio Mixed Use | - | 0.02 | - | 0.33 | 0.05 | Aggregated surface area of mixed land use | - |
| LU Ratio Mobile Homes | - | 0.00 | - | 0.40 | 0.02 | Aggregated surface area of mobile homes land use | - |
| LU Ratio Multi-family | - | 0.08 | 0.05 | 0.69 | 0.11 | Aggregated surface area of multi family land use | - |
| LU Ratio Office | - | 0.09 | 0.06 | 0.57 | 0.10 | Aggregated surface area of office land use | - |
| LU Ratio Open Space and Parks | - | 0.05 | 0.01 | 0.73 | 0.10 | Aggregated surface area of open space and parks land use | - |
| LU Ratio Resource Extraction (Mining) | - | 0.00 | - | 0.19 | 0.01 | Aggregated surface area of resource extraction land use | - |
| LU Ratio Right-of-way | - | 0.00 | - | 0.04 | 0.00 | Aggregated surface area of right-of-way land use | - |
| LU Ratio Single Family | - | 0.06 | 0.00 | 0.50 | 0.09 | Aggregated surface area of single family land use | - |
| LU Ratio Transportation | - | 0.03 | - | 0.73 | 0.11 | Aggregated surface area of transportation land use | - |
| LU Ratio Undeveloped | - | 0.03 | 0.00 | 0.52 | 0.07 | Aggregated surface area of undeveloped land | - |
| LU Ratio Utilities | - | 0.00 | - | 0.07 | 0.01 | Aggregated surface area of utilities land use | - |
| LU Ratio Water | - | 0.00 | - | 0.02 | 0.00 | Aggregated surface area of water bodies | - |
