# Supplementary material for: How urban environment shapes EV charging experience in Travis County, Texas
Source: PLoS One. 2026 Jun 2;21(6):e0349619. doi: 10.1371/journal.pone.0349619 (PMC13229328; doi:10.1371/journal.pone.0349619)
Supplement: Attachment — Submitted filename: Response review.docx [file pone.0349619.s008.docx]

**Review Response to How Urban Environment Shapes EV Charging Experience in Travis**

**Summary of the research**

This Article explores the nature of EV charging stations in Travis County. The article combines serious of methods to perform analysis. The authors use googles images and sentimental reviews to examine each of the charging stations. Additionally, using AI, the authors were able to classify PlugShare reviews into different categories. The article also used the environmental factors from the google images to classify each EVCS according to its derived features. Using random forests, the authors determined the relationships between environmental features and sentimental reviews for each station. The study appears to have conducted a thorough analysis to obtain reasonable results, which are presented in this paper. Additionally, the paper has explored the ongoing technology discussion by integrating it into understanding the EV stations in Travis County.

**Revisions**

**2. Examples and evidence**

1.**Introduction**: Paragraphs one and two should be streamlined to improve the flow.

2.“We then calculated its ratio relative to the total area of its 400m buffer.” This sentence seems dangling for me. Can you make it clearer?

3.“To extract street design elements around each EVCS, we processed each GSV image and

calculated the area that each of the following street design elements occupies: Vegetation, Sky,

Road, Building, Signage, Person, Vehicle, Sidewalk, Fence, and Bench. This value is known

as View Index (VI) and had been used to calculate street design quality indicators such as

complexity and walkability. Additional reference needed in the last paragraph of 3.1

4.Can you organize the figures to match the appearance in the article? If it's possible to rename some of them, some appear as figure 4 while others come as figure4S, etc. Is it possible to have one format through?

5. Is it possible to reduce the wording in the Y axis of Figure 4s(e.g., cut out GSV and mean)? Also, make the font clearer.

**3.Other points (optional)**

1.Clarify if the selected EVCS are primarily public or their private stations.

2.Can you present the method in a graphical view for easy review for the general public, since you have couples of steps entailed in the study.
