# Supplementary material for: How urban environment shapes EV charging experience in Travis County, Texas
Source: PLoS One. 2026 Jun 2;21(6):e0349619. doi: 10.1371/journal.pone.0349619 (PMC13229328; doi:10.1371/journal.pone.0349619)
Supplement: Attachment — Submitted filename: point-to-point-response.docx [file pone.0349619.s009.docx]

**Response to Reviewers**

Title: How Urban Environment Shapes EV Charging Experience in Travis County, Texas

Dear Editor and Reviewers,

We would like to express our sincere gratitude for your constructive, insightful, and comprehensive feedback on our manuscript. We deeply appreciate the time and effort you have invested in reviewing our work. Your comments have been instrumental in refining our conceptual framework, enhancing methodological transparency, and strengthening the academic rigor of our findings.

In accordance with your suggestions, we have undertaken a Major Revision of the manuscript. The key changes include:

- **Methodological Transparency and Data Ethics**: We expanded the "Data overview" section to explicitly detail our data collection processes, confirming that our web scraping and API usage strictly comply with the terms and conditions of all data sources.
- **Commitment to Open Science and Reproducibility**: To fully comply with PLOS ONE’s data and code sharing policies, we have made our final datasets and the author-generated Python code for the Random Forest analysis publicly available without restriction via the OpenICPSR repository.
- **Figure and Visualization Enhancements**: We carefully reviewed all visual elements. To resolve copyright concerns, we clarified the use of open-source OSM data. Furthermore, we refined our data visualizations, ensuring that spatial data is presented via high-quality static image files rather than interactive maps, and updated our scatter plots (e.g., utilizing clear red dots as markers and removing overlapping text) to maximize readability.
- **Formatting and Policy Compliance**: We restructured the manuscript to strictly adhere to PLOS ONE’s style templates. Additionally, we corrected and relocated all funding information to the submission forms (removing it from the Acknowledgments) and properly reclassified our Supporting Information files with appropriate legends.

Below, we provide a detailed, point-by-point response to each comment raised by the Editor and the Reviewers. Our responses are marked in blue, and the corresponding changes made in the manuscript are highlighted accordingly.

Once again, we thank the Editor and the Reviewers for their time, rigor, and invaluable feedback, which has greatly improved the quality of this manuscript. We hope the revised version now meets the high standards of PLOS ONE.

Sincerely,

Corresponding Author

**Editor**

**Comment 1.** Please ensure that your manuscript meets PLOS ONE's style requirements, including those for file naming.

**Response:** We have carefully reviewed PLOS ONE's style requirements and updated the manuscript formatting. This includes adjusting the title page, author affiliations, and headings, as well as updating the file names to strictly adhere to the provided templates.

**Comment 2.** "In your Methods section, please include additional information about your dataset and ensure that you have included a statement specifying whether the collection and analysis method complied with the terms and conditions for the source of the data.

**Response**: Thank you for pointing this out. We have added detailed information regarding the dataset used in this study to the Methods section. Furthermore, we have included a clear statement confirming that our data collection and analysis methods fully comply with the terms and conditions of the data sources (Please see Page 12).

**Comment 3.** Please note that PLOS One has specific guidelines on code sharing for submissions in which author-generated code underpins the findings in the manuscript. In these cases, we expect all author-generated code to be made available without restrictions upon publication of the work.

**Response**: In accordance with PLOS ONE’s code sharing guidelines, we have made the author-generated Python code and the final dataset used for the Random Forest model publicly available without restrictions. The files and related documentation have been permanently deposited in the OpenICPSR repository and can be accessed at: https://www.openicpsr.org/openicpsr/project/246962/version/V1/view

**Comment 4.** We note that the grant information you provided in the ‘Funding Information’ and ‘Financial Disclosure’ sections do not match. When you resubmit, please ensure that you provide the correct grant numbers for the awards you received for your study in the ‘Funding Information’ section.

**Response**: We apologize for the inconsistency in our initial submission. We have verified the information and ensured that the correct grant numbers (NSF Grants 2125858, 2236305) are now provided. As detailed in our response to Comment 5, we have included the unified and accurate Funding Statement in our revised cover letter so that the 'Funding Information' and 'Financial Disclosure' sections can be perfectly aligned in the online submission system.

**Comment 5.** Thank you for stating the following in the Acknowledgments Section of your manuscript:

“This research was supported by the NSF Grants (2125858, 2236305), The MITRE Corporation and UT Good Systems.”

We note that you have provided additional information within the Acknowledgements Section that is not currently declared in your Funding Statement. Please note that funding information should not appear in the Acknowledgments section or other areas of your manuscript. We will only publish funding information present in the Funding Statement section of the online submission form.

Please remove any funding-related text from the manuscript and let us know how you would like to update your Funding Statement. Currently, your Funding Statement reads as follows:

“The author(s) received no specific funding for this work.”

Please include your amended statements within your cover letter; we will change the online submission form on your behalf.

**Response**: Thank you for pointing out this discrepancy. We have removed all funding-related text from the Acknowledgments section or any other areas of the manuscript, as instructed. Furthermore, we have included our amended Funding Statement in our revised cover letter so that the online submission form can be updated on our behalf. The correct funding statement is: "This research was supported by the NSF Grants (2125858, 2236305), The MITRE Corporation and UT Good Systems."

**Comment 6.** We note that Figures 1, 2, S1 & S2 in your submission contain [map/satellite] images which may be copyrighted. All PLOS content is published under the Creative Commons Attribution License (CC BY 4.0), which means that the manuscript, images, and Supporting Information files will be freely available online, and any third party is permitted to access, download, copy, distribute, and use these materials in any way, even commercially, with proper attribution. For these reasons, we cannot publish previously copyrighted maps or satellite images created using proprietary data, such as Google software (Google Maps, Street View, and Earth). For more information, see our copyright guidelines: http://journals.plos.org/plosone/s/licenses-and-copyright.

We require you to either (1) present written permission from the copyright holder to publish these figures specifically under the CC BY 4.0 license, or (2) remove the figures from your submission:

**Response**: Thank you for this observation. We would like to clarify that Figures 1, 2, S1, and S2 do not contain any proprietary or copyrighted data, such as Google Maps or Google Earth. All maps in this submission were generated using OpenStreetMap (OSM) data, which is explicitly recommended by PLOS ONE as a compatible open-source alternative.

To address your concerns and comply with both PLOS ONE and OpenStreetMap’s attribution policies, we have taken the following actions:

1. In-Figure Attribution: We have directly added the required copyright notice—"Map data © OpenStreetMap contributors"—into the bottom-right corner of each figure (Figures 1, 2, S1, and S2) to provide immediate visual confirmation of the data source.
2. Updated Figure Captions: We have updated the legends for these figures to include a direct link to the source and license information: "The base map was created using OpenStreetMap data (https://www.openstreetmap.org/copyright) under a CC BY 4.0 license."
3. License Compatibility: OpenStreetMap data is available under the Open Data Commons Open Database License (ODbL), which is compatible with CC BY 4.0 publication when properly attributed as we have done here.

We confirm that all map content in this manuscript is fully compliant with the CC BY 4.0 license.

**Comment 7.** Please include captions for your Supporting Information files at the end of your manuscript, and update any in-text citations to match accordingly. Please see our Supporting Information guidelines for more information: <http://journals.plos.org/plosone/s/supporting-information>.

**& Comment 8.** We notice that your supplementary figures are uploaded with the file type 'Figure'. Please amend the file type to 'Supporting Information'. Please ensure that each Supporting Information file has a legend listed in the manuscript after the references list.

**Response**: Thank you for the guidance. We have updated the file types for all supplementary figures from 'Figure' to 'Supporting Information' in the online submission system. Additionally, we have ensured that a legend for each Supporting Information file is now clearly listed at the end of the manuscript, immediately following the references list, in accordance with PLOS ONE guidelines.

**Comment 9.** If the reviewer comments include a recommendation to cite specific previously published works, please review and evaluate these publications to determine whether they are relevant and should be cited. There is no requirement to cite these works unless the editor has indicated otherwise.

**Response**: Thank you for this guidance. We have carefully read through all the reviewers' comments and note that there were no specific recommendations to cite previously published works in this instance. We appreciate your helpful clarification regarding citation requirements.

**Reviewer 1**

**Comment 1.1:** Page 5. The authors state, “Furthermore, most existing studies have emphasized model performance and spatial clustering of sentiment, while offering limited insight into the causal mechanisms linking user experience to urban context.” This is a substantial claim and requires supporting references. It is unclear which specific “existing studies” the authors are referencing.

**Response:** We completely agree with the reviewer that this claim needed proper supporting references. In the revised manuscript, we have modified the sentence to be more specific and added the appropriate citations [8, 13] that focus on model performance and spatial clustering without exploring the physical urban context.

**Comment 1.2:** Page 6. The authors should be cautious when stating, “AI has increasingly been used for demand forecasting and system monitoring.” This claim is not adequately supported by the text. While a few experimental case studies are mentioned, the statement implies widespread practitioner adoption, which has not been established.

**Response:** We agree that our original phrasing inadvertently implied widespread practitioner adoption rather than academic exploration. We have revised the sentence in the manuscript to clarify that AI applications in this context are primarily emerging within recent experimental studies. The revised sentence now reads:

*In recent literature, AI-driven approaches have emerged as experimental tools for demand forecasting and system monitoring.*

**Comment 1.3:** Page 7. The statement, “Sentiment analysis of user-generated reviews reveals that perceptions of safety, walkability, and comfort in the charging environment often surface alongside operational feedback,” also requires appropriate citations.

**Response:** We completely agree with the reviewer. To substantiate this statement, we have added appropriate citations to the revised manuscript. Specifically, we cited prior studies that successfully applied machine learning and natural language processing to EV user reviews (e.g., Asensio et al. [6] and Jiao & Chang [8]), which empirically demonstrated that contextual factors such as safety and amenities are heavily discussed alongside operational functionality.

**Comment 1.4:** Section 3.1.1, Study Area. The rationale for selecting Travis County as the study area, rather than the entire Austin Metropolitan Area, is unclear. While the text references the county’s urban core in Austin, it provides little discussion of the suburban and rural portions of the county. Given the heterogeneity of the region, additional explanation is needed to provide adequate contextual grounding.

**Response:** We sincerely thank the reviewer for pointing this out. We agree that the rationale for focusing on Travis County rather than the broader Austin Metropolitan Area (MSA) needed further clarification. In the revised manuscript (Section 3.1.1), we have expanded our explanation to address the county's heterogeneity. We clarified that while the broader MSA presents a highly fragmented policy landscape with sparse EV infrastructure, Travis County offers a distinct urban-to-rural gradient, from Austin’s dense urban core to expanding suburban municipalities and underserved rural peripheries. This contained spatial heterogeneity makes it an ideal, methodologically sound boundary for analyzing how contrasting built environments shape EVCS user experiences.

**Comment 1.5 & 1.6:** The authors should explain why land use, points of interest, economic self-sufficiency, and street design elements were selected as proxy variables rather than alternative measures... The manuscript should also specify which year of American Community Survey data was used and whether the data represent 1 year, 3 year, or 5 year estimates.

**Response:** We sincerely appreciate the reviewer’s constructive suggestions. In the revised Section 3.1.2, we have explicitly justified the selection of our proxy variables. We explained that these proxies were chosen because established urban planning literature demonstrates that macro-level destinations, socio-spatial equity, and micro-level visual comfort are key determinants of user experience and perceived safety in built environments. We have also added appropriate citations to support this rationale.

Furthermore, we have updated the text to specify that the economic self-sufficiency data was derived from the 2024 American Community Survey (ACS) 5-Year Estimates.

**Comment 1.7:** Section 3.2.1. The “structured prompts” used for sentiment analysis should be provided, as this information is necessary for replicability.

**Response:** We completely agree with the reviewer that providing the exact prompts is essential for AI transparency and methodological replicability. In the revised manuscript, we have added a new Supporting Information file (Table S1) that details the exact zero-shot prompts and category-specific questions used to query the generative AI model. We also added a sentence in Section 3.2.1 directing readers to this appendix for full transparency.

**Comment 1.8:** Section 3.2.2. Gemini appears to be treated as a black box that is assumed to be capable of semantic segmentation without sufficient validation by the authors. No related research is cited demonstrating semantic segmentation using Gemini 2.5 Flash. The authors should explain why a more traditional deep learning approach was not considered for this task.

**Response:** We sincerely appreciate the reviewer’s insightful critique regarding the justification and validation of our methodological choice. We agree that the rationale for using Gemini 2.5 Flash over traditional deep learning models needed further elaboration.

In the revised Section 3.2.3 (originally 3.2.2), we have explicitly justified this choice. Traditional deep learning models are highly constrained by a fixed taxonomy. Identifying specific, context-dependent urban elements with these models would require extensive manual annotation and computationally expensive fine-tuning. In contrast, foundational Multimodal Large Language Models (MLLMs) like Gemini 2.5 Flash offer zero-shot, open-vocabulary spatial understanding. This flexibility allows the model to segment objects dynamically based on natural language prompts without specialized retraining.

To address the valid concern regarding validation, we have also added a statement in the manuscript clarifying that we conducted a manual visual inspection of a random subset of 50 Google Street View images. This step verified that Gemini’s generated semantic masks accurately and consistently aligned with human perception for the targeted street elements. We have also added recent literature supporting the use of MLLMs for open-vocabulary segmentation tasks.

**Comment 1.9:** Section 3.2.3. The authors should include a table or figure summarizing the seven random forest regression models and their associated variables. In addition, the choice of random forest regression should be justified relative to alternative modeling approaches.

**Response:** We thank the reviewer for this constructive feedback. We agree that summarizing the models and explicitly justifying our methodological choice improves the clarity of the paper.

In the revised Section 3.2.4 (originally Section 3.2.3), we have added **Table 2**, which clearly outlines the structure of the six models, mapping each dependent variable (sentiment category) to the shared set of independent variables (urban environment proxies). Furthermore, we have expanded the text to explicitly justify the choice of Random Forest over traditional linear models (e.g., OLS regression). We clarified that urban environmental variables often exhibit multicollinearity and non-linear interactions. Random Forest was selected because it is highly robust to these issues and inherently provides reliable feature importance rankings, which aligns directly with our research objective of identifying the most influential environmental factors.

**Comment 1.10:** Finally, the authors should revisit their visualization methods and provide a more thorough explanation of their use of kernel density estimation and hotspot analysis in the methodology section. These techniques may not be familiar to readers outside transportation geography and spatial analysis fields, and additional methodological detail would improve accessibility and transparency.

**Response:** We sincerely thank the reviewer for this excellent suggestion, which greatly improves the clarity and accessibility of our manuscript. Following your recommendation, we have added a detailed explanation of our spatial visualization methods. To ensure logical flow, we introduced a new subsection (**Section 3.2.2 Spatial Pattern Analysis**) before the Generative AI processing and Random Forest sections. In this new section, we provide the mathematical formula for Kernel Density Estimation (KDE) and explain the variables (such as bandwidth and distance) in a clear, non-technical manner. We explicitly describe how this equation transforms discrete, overlapping point data into continuous heat maps that intuitively reveal macro-level urban trends, such as clusters of parking shortages or operational issues. We have also added authoritative citations to support this spatial methodology.

**Comment 1.11:** At a high level, the results provide new and interesting insights into the spatial dimensions of EVCS user experiences that have not been widely explored in prior research. The authors should be commended for this contribution. However, the figures are consistently grainy and difficult to interpret. Higher resolution images with larger labels and added contextual elements, such as network centerlines and municipal boundaries, would greatly improve readability and interpretability.

**Response:** We sincerely appreciate the reviewer’s suggestion to improve the cartographic quality and contextual clarity of our maps.

In the revised manuscript, we have completely regenerated the spatial maps (Figure 2 and Figure 3) using a high-resolution export setting (300 DPI) to ensure excellent visual clarity. To facilitate better geographic interpretation, we have applied a clean, grayscale OpenStreetMap-based basemap (with proper copyright attributions included) which significantly enhances the contrast of the spatial patterns. Furthermore, we overlaid the administrative boundaries of the Austin City Limits and Travis County and added the centerlines of major highways to provide essential spatial reference points. However, to prevent visual clutter and ensure that the Kernel Density hotspots remain the focal point of the maps, we intentionally excluded minor local road centerlines. We believe these cartographic enhancements strike the perfect balance between providing necessary geographic context and maintaining the legibility of our analytical results.

**Comment 1.12:** It is also unclear how “sky” constitutes a meaningful street design element. This variable should likely be removed from the analysis, as the amount of visible sky in Google Street View imagery is unlikely to meaningfully influence user experiences... A more relevant variable would be whether the charging station is covered... Relatedly, an EVCS station does not function in the same way as a bus stop. Many users do not remain at the station while their vehicles charge...

**Response:** We deeply appreciate the reviewer’s exceptional insights regarding the "sky" variable and user behavioral patterns (staying vs. leaving). We completely agree that micro-scale amenities like canopies, as well as the unique behavioral aspect of users leaving their vehicles to utilize nearby amenities, are critical nuances in EVCS research. Rather than removing the "sky" variable, which performed strongly in our models, we have explicitly clarified its urban planning context in the Discussion section (Paragraph 3). We explained that in our macro-level streetscape analysis, the sky variable serves as a proxy for visual openness and the sense of enclosure, which are established urban design elements that influence perceived safety and psychological comfort.

Furthermore, we found the reviewer’s point about user behavior (staying vs. leaving) so compelling that we have integrated it directly into our paper as a key limitation and direction for future research. In the revised Discussion section (Paragraph 5), we explicitly acknowledge that our macro-level GSV variables may not capture micro-scale site designs like canopies. We also explicitly discussed that for users who leave their vehicles to shop or eat, the broader neighborhood amenities take precedence over the immediate visual streetscape.

**Comment 1.13:** Relatedly, an EVCS station does not function in the same way as a bus stop. Many users do not remain at the station while their vehicles charge. Instead, EVCS locations often serve as places where users leave their vehicles while they shop, work, eat, or rest. In this context, the immediate built environment around the station may be of limited relevance to the user experience. This distinction should be explored more explicitly in relation to the study’s findings.

**Response:** We appreciate the reviewer’s exceptional insight regarding EV user behavioral patterns (staying vs. leaving). We completely agree that the unique dwell time of EV charging, where users frequently leave their vehicles to utilize nearby amenities, fundamentally shifts how the built environment should be evaluated. To address this, we have explicitly integrated this behavioral distinction into our interpretation of the findings in the revised Discussion section (Paragraph 4). We clarified that the high predictive importance of macro-level variables like Sidewalks, Walkability, and Commercial Land Use in our Random Forest models directly reflects this "leaving" behavior. Because users leave the station to shop, work, or eat, the immediate environment acts as a pedestrian gateway, making walkability and neighborhood context highly relevant to their overall satisfaction. Furthermore, we explicitly added the nuance of micro-scale immediate environments versus macro-scale neighborhood amenities as a key consideration for future research in the Limitations section. Thank you for providing such a thought-provoking perspective; it has significantly strengthened the logical interpretation of our machine learning results.

**Reviewer 2**

**Comment 2.1:** Introduction: Paragraphs one and two should be streamlined to improve the flow.

**Response:** We thank the reviewer for this constructive feedback. We agree that the transition between the first two paragraphs could be smoother. In the revised manuscript, we have streamlined these paragraphs by removing redundant phrasing regarding the technical role of EVCSs. We also introduced a clearer transitional sentence to better bridge the macro-level urban impacts of EVCSs (Paragraph 1) with the specific research gap concerning human-centered user experiences (Paragraph 2). This revision significantly improves the logical flow and readability of the Introduction.

**Comment 2.2 & 2.3:** “We then calculated its ratio relative to the total area of its 400m buffer.” This sentence seems dangling for me... Additional reference needed in the last paragraph of 3.1 for the View Index (VI).

**Response:** We thank the reviewer for pointing out these areas for improvement. We have revised the dangling sentence to clarify the subject: "We then calculated the ratio of each land use category's area relative to the total area of the corresponding EVCS’s 400m buffer."

Additionally, we have included a relevant reference at the end of Section 3.1 to support the use of the View Index (VI) for calculating street design quality indicators, such as complexity and walkability.

**Comment 2.4:** Clarify if the selected EVCS are primarily public or their private stations.

**Response:** We thank the reviewer for pointing out this distinction. We have clarified in the revised Section 3.1.2. Specifically, we added text explaining that the EVCSs collected in our dataset are primarily publicly accessible stations. We also clarified that while most of these stations are owned and operated by private commercial networks, they are open to the public for use. We excluded restricted residential or workplace-only chargers to focus appropriately on the broader urban charging experience.

**Comment 2.5:** Can you organize the figures to match the appearance in the article? If it's possible to rename some of them, some appear as figure 4 while others come as figure4S, etc. Is it possible to have one format through?

**Response:** We sincerely appreciate the reviewer’s careful attention to the presentation of our manuscript. We completely understand how the different naming conventions might appear inconsistent.

We would like to respectfully clarify that the use of "S" (e.g., Figure S4 or Table S1) denotes "Supporting Information." According to the official PLOS ONE submission guidelines, authors are strictly required to separate main manuscript figures (labeled as Figure 1, Figure 2, etc.) from supplementary figures and to use this specific "S" naming convention for all appendix materials. Therefore, to ensure full compliance with the journal's formatting policies, we must maintain this distinction between the main text figures and the Supporting Information figures. We hope this explanation clarifies the formatting choice.

**Comment 2.6:** Is it possible to reduce the wording in the Y axis of Figure 4s (e.g., cut out GSV and mean)? Also, make the font clearer.

**Response:** We completely agree with the reviewer that the original y-axis labels in Figure S4 (which included raw data suffixes like 'GSV' and 'mean') were unnecessarily long and hindered readability.

Following your helpful suggestion, we have completely regenerated Figure S4. We cleaned and abbreviated all variable names (e.g., changing "GSV_Sidewalk_mean" to "Sidewalk" and "Ratio of Income to Poverty Level" to "Poverty Ratio"). Additionally, we significantly increased the font sizes for the titles, axis labels, and tick marks to ensure the figure is clear and easy to read. These changes have greatly improved the visual quality and readability of the results.

**Comment 2.7:** Can you present the method in a graphical view for easy review for the general public, since you have couples of steps entailed in the study.

**Response:** We sincerely thank the reviewer for this excellent and encouraging suggestion. We agree that a visual representation of our complex method is essential for a general audience. We have created a simplified, high-level methodological flowchart and added it as **Figure 1** in the revised manuscript. This visual diagram clearly illustrates the four-phase analytical pipeline, from data collection and simplified AI processing to spatial integration and machine learning modeling, enabling readers to grasp the core methodological logic at a single glance. Thank you for helping to improve the accessibility of our work.
